# Temporal trend of food intake markers among adults recorded on the Food and Nutrition Surveillance System, Brazil: time series analysis

**DOI:** 10.1590/S2237-96222025v34e20240041.en

**Published:** 2025-06-13

**Authors:** Anael Queirós Silva Barros, Ítalo Wesley Oliveira de Aguiar, Antonio Augusto Ferreira Carioca, Lia Silveira Adriano, Helena Alves de Carvalho Sampaio

**Affiliations:** 1 Universidade Estadual do Ceará, Programa de Pós-Graduação em Saúde Coletiva, Fortaleza, CE, Brazil; 2 Universidade Federal do Ceará, Programa de Pós-Graduação em Saúde Pública, Fortaleza, CE, Brazil; 3 Universidade de Fortaleza, Programa de Pós-Graduação em Saúde Coletiva, Fortaleza, CE, Brazil

**Keywords:** Nutritional Epidemiology, Primary Health Care, Health Information Systems, Time Series Studies, Epidemiología Nutricional, Atención Primaria de Salud, Sistemas de Información en Salud, Estudios de Series Temporales

## Abstract

**Objectives:**

To analyze the temporal trend of food intake markers of adults recorded on the Food and Nutrition Surveillance System in Brazil and its macro-regions in the period 2015-2019 and to compare food intake from 2015-2019 with that from 2020-2023.

**Methods:**

This is a time series analysis study, based on secondary data of adults (20-59 years old) held on the Food and Nutrition Surveillance System online platform, referring to the period 2015-2019, and a comparative study of the periods 2015-2019 and 2020-2023, analyzing Brazil and its five macro-regions. Prevalence of food intake (healthy or unhealthy) on the previous day was verified. Intake of beans, fresh fruit, vegetables and legumes was considered healthy behavior; while intake of hamburgers and sausages, sweetened beverages, instant noodles, packaged snacks or savory biscuits, and intake of stuffed cookies, sweets or treats was considered unhealthy behavior. Temporal trends were estimated by percentage change in the period and a Prais-Winsten regression model.

**Results:**

The records of 7,549,781 adults were analyzed. In Brazil as a whole, a temporal trend of growth in intake of hamburgers and sausages (4.5%) and fresh fruit (1.4%) and a reduction in sweetened beverages (-1.2%) was found. The comparison, made between the periods 2015-2019 and 2020-2023, pointed to possible changes in food intake due to the COVID-19 pandemic, with an increase in intake of hamburgers and sausages (18.8%) and instant noodles (5.8%).

**Conclusion:**

There was an increase in intake of unhealthy foods by the Brazilian adult population, with regional disparities and impacted by the pandemic.

## Resumen

**Objetivos:**

Analizar la tendencia temporal de los marcadores de consumo de alimentos de los adultos registrados en el Sistema de Vigilancia Alimentaria y Nutricional en Brasil y en sus macrorregiones en el período 2015-2019 y comparar el consumo de 2015-2019 con el de 2020-2023.

**Métodos:**

Se trata de un estudio de análisis de series temporales, basado en datos secundarios de adultos (20-59 años) de la plataforma en línea del Sistema de Vigilancia Alimentaria y Nutricional, referidos al período 2015-2019, y un estudio comparativo de los períodos 2015-2019 y 2020-2023, analizando Brasil y sus cinco macrorregiones. Se verificó la prevalencia del consumo de alimentos (saludables o no saludables) del día anterior. Se consideró comportamiento saludable el consumo de frijoles, frutas, verduras y legumbres frescas; se consideraron no saludables las hamburguesas y embutidos, las bebidas azucaradas, los fideos instantáneos, los snacks envasados ​​o las galletas saladas y el consumo de galletas rellenas, dulces o golosinas. Las tendencias temporales se estimaron utilizando el cambio porcentual durante el período y el modelo de regresión de Prais-Winsten.

**Resultados:**

Se analizaron los expedientes de 7.549.781 adultos. En Brasil, hubo una tendencia temporal de crecimiento en el consumo de hamburguesas y embutidos (4,5%) y de frutas frescas (1,4%) y de reducción en el de bebidas azucaradas (-1,2%). La comparación, realizada entre los períodos 2015-2019 y 2020-2023, destacó posibles cambios en el consumo de alimentos debido a la pandemia de Covid-19, con un aumento en el consumo de hamburguesas y embutidos (18,8%) y fideos instantáneos (5,8%).

**Conclusión:**

Hubo un aumento en el consumo de alimentos no saludables por parte de la población adulta brasileña con disparidades regionales e impactada por la pandemia.

**Ethical aspects**

This research respected ethical principles, having obtained the following approval data:

Research Ethics Committee: Universidade de Fortaleza

Opinion number: 4,348,452

Approval date: 20/10/2020

Certificate of Submission for Ethical Appraisal: 31540320.9.1001.5052

Informed Consent Form: Exempted.

## Introduction

The Food and Nutrition Surveillance System is a national health information system that operates as a food and nutrition surveillance tool. This system manages, monitors and plans actions regarding the food and nutrition status of individuals who use the Brazilian National Health System (*Sistema Único de Saúde* - SUS) (1).

In accordance with the Dietary Guidelines, with effect from 2015 (2,^3^), the system has been evaluating food intake markers (4), through a marker-type survey (5), which investigates the intake of certain foods, such as beans, fresh fruit and vegetables, classified as healthy, and foods, such as hamburgers and sausages, sweetened beverages, instant noodles, packaged snacks, savory biscuits, stuffed cookies, sweets and treats, considered unhealthy, as they are in the processed and ultra-processed food category (2).

The food marker form can be administered quickly, practically and effectively, showing the food and nutritional scenario of the Brazilian population monitored in primary care through the SUS (4). The instrument has the potential to monitor temporal trends, generating information for guidance, decision-making, formulation and evaluation of public policies for health promotion and prevention (6,7).

The system has become an essential tool for monitoring temporal trends in food intake and assessing the impacts of these changes on public health. Analyzing food intake trends in the periods 2015-2019 and 2020-2023, considering regional disparities, is essential for understanding how the health crisis influenced the population’s diet. This has contributed to the formulation of more effective public policies, focusing on promoting health and reducing food and nutritional inequalities. The objective of this study was to analyze the temporal trend of food intake markers of adults registered on the Food and Nutrition Surveillance System in Brazil and in the country’s macro-regions between 2015 and 2019, and to compare food intake in 2015-2019 with food intake in 2020-2023.

## Methods

### Design 

This is a time series analysis study, based on annual secondary data available on the Food and Nutrition Surveillance System online platform, web version, for the period 2015-2019, and a comparative study of the period 2015-2019 with 2020-2023, analyzing Brazil and its five macro-regions. 

### Setting

The data analyzed were taken from the public access reports (8), available at https://sisaps.saude.gov.br/sisvan/relatoriopublico/index, with records related to food intake markers of adults in the periods 2015-2019 and 2020-2023. 

The first version, named Food and Nutrition Surveillance System - Municipal Module, was introduced through Ordinance No. 2,246, dated October 18, 2004 (9). In 2008, the new version was released as the Food and Nutrition Surveillance System - Web version. In 2015, the new food intake assessment forms became available (4). In 2017, version 3.0 of the system was launched (10), with the aim of optimizing the integration of the Food and Nutrition Surveillance System with the e-SUS Primary Care (*e-SUS Atenção Básica*) system, as per the Ministry of Health technical note (11). 

The period 2015-2019 was chosen because 2015 was the year in which the new food intake forms were introduced, and 2019 was the last year before the start of the COVID-19 pandemic. This period was chosen considering that, until 2019, no disease control and prevention measures had been implemented that are known to have influenced daily habits and regular access to health services. It is assumed that the data for this period represent the usual profile of the target population.

In addition to identifying the temporal trend of annual values ​​between 2015 and 2019, food intake was investigated in the period before and after the start of the pandemic, through comparisons between the aggregate food intake percentages in the periods 2015-2019 and 2020-2023.

### Participants

We analyzed data aggregated by geographic region (the country’s macro-regions and Brazil as a whole), for adult individuals aged 20-59 years of both sexes, based on data available on the Food and Nutrition Surveillance System - Web version.

### Variables 

The new Food and Nutrition Surveillance System food intake marker forms, introduced in 2015, were developed based on the new version of the Dietary Guidelines. They covered the food intake of children over 2 years of age, adolescents, adults, the elderly and pregnant women, and assessed food items consumed on the previous day, which helped to minimize possible memory biases (2-4). 

The study analyzed previous day food intake markers (healthy or unhealthy). Intake of beans, fresh fruit, vegetables and legumes was considered healthy; and unhealthy behavior was considered to be intake of hamburgers and sausages, sweetened beverages, instant noodles, packaged snacks or savory biscuits, as well as intake of stuffed cookies, sweets or treats (4).

### Data sources and measurement 

The data were extracted from the Food and Nutrition Surveillance System platform, accessed at https://sisaps.saude.gov.br/sisvan/relatoriopublico/index. The platform contained annual public reports, which included all types of recorded monitoring, originating from the system itself, from the Bolsa Família Program management system and from the e-SUS Primary Health Care system (8). For this study, we extracted food intake data from the last assessment of the year for each adult individual, which were collected on the website mentioned above and exported in Excel spreadsheet format on March 20, 2024. 

### Bias control

In order to reduce the risk of bias, Food and Nutrition Surveillance System protocols were applied a priori to exclude implausible values. 

### Study size

We analyzed data aggregated by geographic region (the country’s macro-regions and Brazil as a whole), comprising 7,549,781 registered adults. The Food and Nutrition Surveillance System coverage percentage was calculated by the annual number of records, divided by the population exclusively using the SUS, multiplied by 100. The population exclusively using the SUS was estimated based on the number of Brazilians aged 20-59 (12), subtracted from the number of Brazilians in this age group who were beneficiaries of private health plans with medical assistance in June of each year between 2015 and 2023, according to data from the National Supplementary Health Agency (13).

### Statistical methods

Percentage change in each food intake marker in the period between 2015 and 2019 was calculated by applying the following equation: “((Value in 2019 / Value in 2015) - 1) x 100”. A similar calculation was applied between 2020 and 2023. Association between increase in a year and food intake as per the markers was investigated using the Prais-Winsten regression model, in which the proportion of intake of the markers was used as the dependent variable. This variable underwent logarithmic transformation, and the resulting beta coefficients were subjected to an exponential function. Annual percentage change was calculated using the equation “(1 - e^β^) x 100”.

The values ​​for 2020 to 2023 were compared with the values ​​for 2015 to 2019, based on the calculation of percentage change. This calculation consisted of subtracting the percentages for 2020 to 2023 from the percentages for 2015 to 2019, dividing by the percentage for 2020 to 2023, and multiplying the result by 100. Comparison of these periods was performed using Prais-Winsten regression. Positive or negative annual percentage changes indicated rising or falling trends.

For all analyses, p-value<0.05 was considered significant. Statistical analyses were performed using Stata Statistical software: version 17 (College Station, TX: StataCorp LLC), using the “Prais” library (14).

### Data access and cleaning methods

The process undertaken in this research met National Health Council standards, as well as the standards and instructions contained in Ordinance No. 884, dated December 13, 2011 (15). The project was approved by the *Universidade de Fortaleza* Research Ethics Committee. All records available on the system for the age, temporal and spatial groups in question were used in this analysis. 

### Data matching

For this study, there was no need for data linkage at any level or database. We used data from the Food and Nutrition Surveillance System - Web version. 

## Results

Between 2015 and 2023, 7,549,781 adults aged 20-59, of both sexes, were recorded by the system. Annual coverage of the Food and Nutrition Surveillance System at the national level showed the lowest percentage in 2015 (0.2%). The year 2019 (1.0%) marked the increase in this coverage. In 2020, there was a decrease (0.8%), followed by an increase with a rising trend in 2021 (1.2%), 2022 (1.8%) and 2023 (3.1%) (Table 1).

**Table 1:** Percentage national annual coverage of adults recorded on the Food and Nutrition Surveillance System. Brazil, 2015-2023

Year	(%)
2015	0.2
2016	0.4
2017	1.0
2018	0.8
2019	1.0
2020	0.8
2021	1.2
2022	1.8
2023	3.1

Annual percentage change in intake of beans remained stable in Brazil and its macro-regions between 2015 and 2019. In those five years, the percentage of people who consumed fresh fruit the previous day in Brazil grew, on average, by 1.4% per year (95% confidence interval [95%CI] 0.3; 2.6). Intake of hamburgers and sausages grew by 4.5% per year (95%CI 2.3; 6.7), while there was a 1.2% decrease for sweetened beverages (95%CI -1.8; -0.6). In the Northern region, there was a 4.5% reduction per year in intake of sweetened beverages (95%CI -6.8; -2.3) and a 6.4% reduction (95%CI -12.1; -0.3) in intake of stuffed cookies, sweets or treats (Table 2).

**Table 2:** Annual percentage change and 95% confidence intervals (95%CI) of the Food and Nutrition Surveillance System food intake markers. Brazil and geographic regions, 2015-2019

Markers	Percentage change in the period (%)^a^	Annual percentage change^b^		
		(%)	(95%CI)	Trend
Brazil				
Beans	0.0	0.2	(-0.4; 0.8)	Stable
Fresh fruit	2.8	1.4	(0.3; 2.6)	Rising
Vegetables and legumes	4.1	1.5	(-0.7; 3.7)	Stable
Hamburger and sausages	15.6	4.5	(2.3; 6.7)	Rising
Sweetened beverages	-5.3	-1.2	(-1.8; -0.6)	Falling
Instant noodles etc.	-5.7	-0.2	(-5.9; 5.7)	Stable
Stuffed cookies etc.	-2.8	0.1	(-1.1; 1.3)	Stable
North				
Beans	-6.3	-0.2	(-2.3; 2.0)	Stable
Fresh fruit	-4.2	-0.4	(-1.7; 0.9)	Stable
Vegetables and legumes	0.0	0.5	(-0.8; 1.9)	Stable
Hamburger and sausages	-6.9	1.2	(-5.6; 8.4)	Stable
Sweetened beverages	-17.0	-4.5	(-6.8; -2.3)	Falling
Instant noodles etc.	-23.0	-4.8	(-13.3; 4.6)	Stable
Stuffed cookies etc.	-25.0	-6.4	(-12.1; -0.3)	Falling
Northeast				
Beans	0.0	0.1	(-1.1; 1.4)	Stable
Fresh fruit	2.8	1.5	(-0.6; 3.6)	Stable
Vegetables and legumes	9.1	3.0	(1.4; 4.5)	Rising
Hamburger and sausages	3.3	1.6	(-7.3; 11.4)	Stable
Sweetened beverages	-2.0	-0.6	(-5.4; 4.5)	Stable
Instant noodles etc.	-10.5	-2.0	(-6.9; 3.1)	Stable
Stuffed cookies etc.	3.2	1.1	(-0.0; 2.2)	Stable
Midwest				
Beans	3.4	1.1	(-2.1; 4.5)	Stable
Fresh fruit	-1.3	0.4	(-2.0; 2.8)	Stable
Vegetables and legumes	3.8	1.2	(0.3; 2.2)	Rising
Hamburger and sausages	20.6	5.0	(-4.4; 15.4)	Stable
Sweetened beverages	-1.7	-0.1	(-6.3; 6.4)	Stable
Instant noodles etc.	-13.9	-2.9	(-15.6; 11.8)	Stable
Stuffed cookies etc.	-12.2	-3.1	(-6.4; 0.4)	Stable
Southeast				
Beans	2.3	0.7	(-0.3; 1.8)	Stable
Fresh fruit	2.9	1.5	(0.4; 2.6)	Rising
Vegetables and legumes	2.6	0.8	(-0.3; 2.0)	Stable
Hamburger and sausages	18.2	3.9	(1.2; 6.7)	Rising
Sweetened beverages	-8.2	-1.4	(-2.4; -0.4)	Falling
Instant noodles etc.	-3.1	0.6	(-1.4; 2.6)	Stable
Stuffed cookies etc.	-7.7	-2.2	(-2.9; -1.6)	Falling
South				
Beans	-2.5	-1.1	(-3.6; 1.5)	Stable
Fresh fruit	4.1	0.9	(-3.0; 5.0)	Stable
Vegetables and legumes	-1.2	-0.4	(-3.5; 2.9)	Stable
Hamburger and sausages	17.8	4.6	(-2.5; 12.3)	Stable
Sweetened beverages	-1.6	-1.1	(-1.2; -1.1)	Falling
Instant noodles etc.	9.4	3.8	(1.0; 6.6)	Rising
Stuffed cookies etc.	11.1	3.7	(1.9; 5.5)	Rising

Notes: ^a^Percentage change in food intake markers in the period 2015-2019; ^b^Annual percentage change in food intake markers.

Between 2015 and 2019, in the Northeast region, the percentage of people who consumed vegetables and legumes the previous day grew, on average, by 3.0% per year (95%CI 1.4; 4.5). In the Southeast region, the percentage of people recorded on the system who consumed fruit the previous day grew, on average, by 1.5% per year (95%CI 0.4; 2.6). Intake of hamburgers and sausages the previous day also grew, being 3.9% per year (95%CI 1.2; 6.7). There was a decrease of 1.4% in sweetened beverages (95%CI -2.4; -0.4) and a decrease of 2.2% (95%CI -2.9; -1.6) per year in stuffed cookies, sweets and treats (Table 2). 

In the Southern region, between 2015 and 2019, the percentage of people who consumed sweetened beverages on the previous day decreased, on average, by 1.1% per year (95% CI -1.2; -1.1). Intake of instant noodles, packaged snacks or savory biscuits increased, on average, by 3.8% per year (95% CI 1.0; 6.6), and consumption of stuffed cookies, sweets and treats increased by 3.7% per year (95% CI 1.9; 5.5). In the Midwest, the percentage of people who consumed vegetables and legumes on the previous day increased, on average, by 1.2% per year (95% CI 0.3; 2.2) between 2015 and 2019 (Table 2).

Comparing the two periods, there was statistical evidence of growth in markers of unhealthy food intake in all macro-regions and in Brazil as a whole in the period 2020-2023, when compared to 2015-2019. In the North, Northeast, Midwest, and Southeast regions and in Brazil as a whole, there was an increase in intake of hamburgers and sausages. In the Midwest, Southeast, and Southern regions and in Brazil as a whole, there was an increase in intake of instant noodles, packaged snacks, and savory biscuits. In Brazil as a whole and in the Northern, Northeast, Southern, and Southeast regions, there was no increase or reduction in intake of the other unhealthy foods. In the Midwest, there was an increase in intake of sweetened beverages and stuffed cookies, sweets, and treats (Table 3).

**Table 3:** Percentage and percentage change of the Food and Nutrition Surveillance System food intake markers. Brazil and geographic regions, 2015-2019 and 2020-2023

Markers	Percentage in the period 2015-2019	Percentage in the period 2020-2023	Percentage change between the periods	p-value
Brazil				
Beans	85.3	80.2	-6.0	0.142
Fresh fruit	73.0	71.4	-2.3	0.365
Vegetables and legumes	76.2	79.1	3.8	0.072
Hamburger and sausages	35.6	42.3	18.8	0.006
Sweetened beverages	54.6	54.8	0.5	0.620
Instant noodles etc.	32.1	34.0	5.8	0.041
Stuffed cookies etc.	35.0	36.4	3.9	0.116
North				
Beans	76.4	75.5	-1.2	0.290
Fresh fruit	68.3	73.0	6.8	0.979
Vegetables and legumes	65.6	76.2	16.2	0.003
Hamburger and sausages	26.5	36.1	36.0	<0.001
Sweetened beverages	52.0	51.7	-0.5	0.863
Instant noodles etc.	31.5	31.2	-1.2	0.648
Stuffed cookies etc.	31.8	32.6	2.5	0.868
Northeast				
Beans	88.9	84.7	-4.7	0.154
Fresh fruit	72.9	74.3	1.9	0.872
Vegetables and legumes	69.4	76.1	9.7	0.005
Hamburger and sausages	28.6	37.0	29.2	0.003
Sweetened beverages	48.3	48.9	1.3	0.493
Instant noodles etc.	33.9	34.1	0.6	0.766
Stuffed cookies etc.	31.1	32.3	3.9	0.062
Midwest				
Beans	87.9	82.3	-6.4	0.211
Fresh fruit	72.8	70.0	-4.5	0.252
Vegetables and legumes	80.7	82.8	2.7	0.035
Hamburger and sausages	35.4	47.2	33.2	0.002
Sweetened beverages	55.9	61.0	9.1	0.021
Instant noodles etc.	28.6	34.8	21.6	0.020
Stuffed cookies etc.	35.1	40.9	16.4	0.019
Southeast				
Beans	87.9	85.3	-3.0	0.292
Fresh fruit	71.8	72.3	0.7	0.629
Vegetables and legumes	80.0	83.9	5.0	0.002
Hamburger and sausages	37.4	44.7	19.5	0.001
Sweetened beverages	57.7	58.8	1.9	0.601
Instant noodles etc.	31.3	33.4	6.6	0.025
Stuffed cookies etc.	36.8	38.5	4.6	0.276
South				
Beans	78.5	61.5	-21.7	0.041
Fresh fruit	79.5	61.9	-22.2	0.062
Vegetables and legumes	81.2	75.1	-7.6	0.020
Hamburger and sausages	47.9	52.3	9.3	0.091
Sweetened beverages	58.4	57.7	-1.2	0.908
Instant noodles etc.	33.2	37.4	12.8	0.007
Stuffed cookies etc.	39.2	41.6	6.2	0.053

There was no change in Brazil as a whole in relation to healthy food intake markers, but in the analysis stratified by regions, there was a significant increase in vegetables/legumes in all regions, except in the Southern region, where there was a reduction. In the Southern region, there was a significant reduction in intake of beans (Table 3).

## Discussion

This study analyzed the temporal trend of food intake of adults recorded on the Food and Nutrition Surveillance System in the period 2015-2019 and compared it to 2020-2023. For Brazil as a whole we found a temporal trend of growth in intake of hamburgers and sausages and fresh fruit and a reduction in sweetened beverages. The comparison between the periods pointed to possible changes in food consumption due to the COVID-19 pandemic, with an increase in intake of hamburgers and sausages and instant noodles.

The choice of markers as an analysis instrument allowed us to identify intake of healthy and unhealthy foods by adults who are primary care users and who are treated by the SUS in Brazil and its regions. Healthy markers include intake of beans, fresh fruit, vegetables and legumes; while unhealthy markers are related to intake of hamburgers and sausages, sweetened beverages, instant noodles, packaged snacks or savory biscuits, as well as stuffed cookies, sweets or treats (4). This instrument has become a tool for identifying health conditions, guiding eating habits and portraying weaknesses associated with diet, working effectively and objectively as markers (5).

The results found should be interpreted considering certain limitations. As this is an ecological study, it may be biased due to causal inferences. Considering the quality of data held on health information systems, from a multidimensional perspective, the coverage of the data entered was assessed, as it is one of the most evaluated dimensions in the Food and Nutrition Surveillance System and other systems (16). Although it presents modest coverage compared to anthropometric data, there is a consistent growth profile (17). The forms assess the foods that were consumed the previous day, mitigating memory biases, with objective, clear and easy-to-administer questions. This instrument is recommended by the Ministry of Health. There is no information on usual amounts or frequencies.

Among the coverage percentages at the national level, the lowest percentage found for in 2015 was expected, as it marked the beginning of the implementation of the new food intake forms. Between 2018 and 2019, there was a notable increase in coverage, as data entry began in 2017 via the e-SUS Primary Care system, to the detriment of the Food and Nutrition Surveillance System - Web version. The year 2019 was marked as the last before the start of the COVID-19 pandemic, with the best percentage among previous years. In 2020, percentage coverage declined. The years following the pandemic continued to see growth in coverage.

The system has been improved and updated over the years, improving its coverage and data quality. Total population coverage was considered preliminary, presenting some limitations as to the representativeness of the data. Progress in system coverage and a rising trend following data entry via the e-SUS Primary Care system have been noted over the years (18,19).

Comparing the Brazilian regions between 2015 and 2019, both at the national and macro-regional level, a stable change in the annual percentage of the beans food intake marker was identified. There was greater growth in the percentage of healthy markers in the Northeast region for vegetables and legumes. The greatest decrease in unhealthy eating occurred in the Northern region. The greatest percentage increase in unhealthy foods was found for the Midwest region, in the proportion of foods related to processed meats, such as hamburgers and sausages.

Intake of fruit, vegetables and legumes was protective against cardiovascular diseases, and daily intake of these foods is recommended (3). The frequency of adults who regularly consumed fruit and vegetables was considered stable between 2008 and 2021, ranging from 33.0% in 2008 to 34.2% in 2021 (20).

The processed and ultra-processed foods most consumed by the sample in this study between 2015 and 2019 were hamburgers and sausages in Brazil as a whole and in the Southeast region, and instant noodles, packaged snacks or savory biscuits and stuffed cookies, sweets or treats in the Southern region. The decrease observed in the percentage of people in Brazil as a whole and in the Northern, Southeast and Southern regions who consumed sweetened beverages was in line with previous data (21,22).

In Brazil as whole, there was a greater share of ultra-processed foods in the diet, being related to health problems in the adult population, which characterized the scenario regarding the nutritional profile (23-25). It has been emphasized that use of markers, at an individual and population level, has made it possible to recognize healthy and unhealthy eating habits, in addition to allowing monitoring of the quality of the diet of the entire Brazilian population (6,7).

This study found that, between the period 2015-2019 and the period 2020-2023, there was an increase in unhealthy markers in all macro-regions and in Brazil as a whole. The COVID-19 pandemic changed the population’s eating habits, with a reduction in intake of unprocessed and minimally processed foods and an increase in ultra-processed foods (26,27). Clear associations have been found between socioeconomic and lifestyle variables and inadequate eating habits (26) along with stress, anxiety, and depression behaviors during the pandemic period (28,29).

Between 2015-2019 and 2020-2023, among healthy foods, the highest percentage changes stood out for vegetables and legumes in the Northeast (+9.7%) and Northern (+16.2%) regions, and for unhealthy foods in the Midwest region: hamburgers and sausages (+33.2%), sweetened beverages (+9.1%), instant noodles, packaged snacks or savory biscuits (+21.6%) and stuffed cookies, sweets or treats (+16.4%). There was an increase in ultra-processed foods among the Brazilian adult population during the COVID-19 pandemic (26,27).

Bean consumption did not vary in the two time periods, with the exception of the Southern region, where there was a reduction. This data differed from information obtained previously. This indicated that the frequency of adults, between 2007 and 2021, who reported eating beans on five or more days a week decreased, ranging from 66.8% in 2007 to 60.4% in 2021 (20). Data from the Household Budget Survey in 2008-2009 (30) and 2017-2018 (22) also portrayed the decline of beans in the Brazilian diet.

It can be understood that the food intake of adult primary care users in Brazil has regional disparities, accentuating the reflection of climatic characteristics, economic activities, geographic access, diversity of food cultures, in addition to social inequalities between regions (18,19). This contributes to changes in dietary patterns, associated with the healthy or unhealthy behaviors found in this study.

The system has contributed to the monitoring and management of Food and Nutrition Surveillance information, in accordance with the guidelines of the National Food and Nutrition Policy (31). The information obtained from the markers covers the diagnosis and surveillance of the dietary conditions of the adult population, which assesses the effectiveness and efficiency of public policies (6,16). The use of secondary data, analyzed through the Food and Nutrition Surveillance System, is modest in this population, constituting a large area for future research.

We conclude that there was an increase in the annual coverage of adult individuals in Brazil with records of food intake markers, which reached 1.0% of the population in the pre-pandemic period in 2019. There was a decrease in this coverage in the following year and an increase in subsequent years. 

The results found identified progressively unhealthy national food intake in the period 2015-2019, mainly with the presence of increased consumption of hamburgers and sausages and instant noodles, packaged snacks or savory biscuits. This trend can be offset by the increased presence of vegetables/legumes in the food eaten. The comparison made between the periods 2015-2019 and 2020-2023 pointed out possible changes in food intake due to the COVID-19 pandemic.

## Data Availability

The database used in this research is available at: https://figshare.com/s/6337256d2548a3f0ed5f.
