# Erratum: “Temporal trend of food intake markers among adults recorded on the Food and Nutrition Surveillance System, Brazil: time series analysis”

**DOI:** 10.1590/S2237-96222025v34e20240041e.en

**Published:** 2026-08-10

In the article “Temporal trend of food intake markers among adults recorded on the Food and Nutrition Surveillance System, Brazil: time series analysis”, doi 10.1590/S2237-96222025v34e20240041.en, published on *Epidemiol Serv Saude*. 2025:34;e20240041, on page 10.

**Original text**

"The database used in this research is available at: https://figshare.com/s/6337256d2548a3f0ed5f."

**Corrected text**

"The database used in this research is available at: https://sisaps.saude.gov.br/sisvan/relatoriopublico/index."

