# Supplementary material for: Temporal trend of food intake markers among adults recorded on the Food and Nutrition Surveillance System, Brazil: time series analysis
Source: Epidemiol Serv Saude. 2025 Jun 13;34:e20240041. doi: 10.1590/S2237-96222025v34e20240041.en (PMC12176433; doi:10.1590/S2237-96222025v34e20240041.en)
Supplement: Translation

# Translation: Tendência temporal de marcadores do consumo alimentar entre adultos registrados no Sistema de Vigilância Alimentar e Nutricional, Brasil: análise de série temporal

**DOI:** 10.1590/S2237-96222025v34e20240041.pt

**Authors:** Anael Queirós Silva Barros, Ítalo Wesley Oliveira de Aguiar, Antonio Augusto Ferreira Carioca, Lia Silveira Adriano, Helena Alves de Carvalho Sampaio

**Affiliations:** 1 Universidade Estadual do Ceará, Programa de Pós-Graduação em Saúde Coletiva, Fortaleza, CE, Brasil; 2 Universidade Federal do Ceará, Programa de Pós-Graduação em Saúde Pública, Fortaleza, CE, Brasil; 3 Universidade de Fortaleza, Programa de Pós-Graduação em Saúde Coletiva, Fortaleza, CE, Brasil

**Keywords:** Epidemiologia Nutricional, Atenção Primária à Saúde, Sistemas de Informação em Saúde, Estudos de Séries Temporais

## Abstract

**Objetivos:**

Analisar a tendência temporal dos marcadores do consumo alimentar de adultos cadastrados no Sistema de Vigilância Alimentar e Nutricional no Brasil e nas macrorregiões no período 2015-2019 e comparar o consumo de 2015-2019 com o de 2020-2023.

**Métodos:**

Trata-se de estudo de análise de série temporal, com base nos dados secundários de adultos (20-59 anos) da plataforma online do Sistema de Vigilância Alimentar e Nutricional, referente ao período 2015-2019, e estudo comparativo dos períodos 2015-2019 e 2020-2023, analisando o Brasil e suas cinco macrorregiões. Verificou-se a prevalência do consumo alimentar (saudáveis ou não saudáveis) do dia anterior. Consideraram-se como comportamento saudável o consumo de feijão, frutas frescas, verduras e legumes; e não saudável o de hambúrguer e embutidos, bebidas adoçadas, macarrão instantâneo, salgadinhos de pacote ou biscoitos salgados e consumo de biscoitos recheados, doces ou guloseimas. Tendências temporais foram estimadas por variação percentual no período e modelo de regressão de Prais-Winsten.

**Resultados:**

Analisaram-se os registros de 7.549.781 adultos. No Brasil, observou-se tendência temporal de crescimento do consumo de hambúrguer e embutidos (4,5%) e frutas frescas (1,4%) e redução de bebidas adoçadas (-1,2%). A comparação, realizada entre os períodos 2015-2019 e 2020-2023, apontou possíveis modificações do consumo alimentar devido à pandemia de covid-19, com aumento no consumo de hambúrguer e embutidos (18,8%) e macarrão instantâneo (5,8%).

**Conclusão:**

Houve aumento do consumo de alimentos não saudáveis pela população adulta brasileira com disparidades regionais e impactado pela pandemia.

**Aspectos éticos**

A presente pesquisa respeitou os princípios éticos, obtendo os seguintes dados de aprovação: :

Comitê de ética em pesquisa: Universidade de Fortaleza

Número do parecer: 4.348.452

Data de aprovação: 20/10/2020

Certificado de apresentação de apreciação ética: 31540320.9.1001.5052

Registro do consentimento livre e esclarecido: Dispensado.

## Introdução

O Sistema de Vigilância Alimentar e Nutricional trata-se de um sistema de informação em saúde, a nível nacional, que opera como ferramenta de vigilância alimentar e nutricional. Esse sistema exerce a funcionalidade de gerenciar, monitorar e planejar ações acerca da situação alimentar e nutricional de indivíduos que são atendidos pelo Sistema Único de Saúde (SUS) (1).

Em concordância com o guia alimentar, a partir de 2015 (2,3), o sistema tem realizado a avaliação de marcadores do consumo alimentar (4), por meio de inquérito do tipo marcadores (5), que investiga a ingestão de alguns alimentos, como feijão, frutas frescas e verduras/legumes, classificados como saudáveis, e de alimentos, como hambúrguer e embutidos, bebidas adoçadas, macarrão instantâneo, salgadinhos de pacote, biscoitos salgados, biscoitos recheados, doces e guloseimas, considerados não saudáveis, pois incluem alimentos processados e ultraprocessados (2).

O formulário com os marcadores alimentares pode ser aplicado de forma rápida, prática e eficaz, mostrando o cenário alimentar e nutricional da população brasileira acompanhada na atenção primária por meio do SUS (4). O instrumento apresenta potencial para acompanhar as tendências temporais, gerando subsídios para orientações, tomada de decisões, formulações e avaliações de políticas públicas de promoção e prevenção à saúde (6,7).

O sistema se tornou ferramenta essencial para monitorar as tendências temporais do consumo alimentar e avaliar os impactos dessas mudanças na saúde pública. A análise das tendências de consumo nos períodos 2015-2019 e 2020-2023, considerando as disparidades regionais, é fundamental para entender como a crise sanitária influenciou a alimentação da população. Isso contribuiu para a formulação de políticas públicas mais eficazes, com foco na promoção da saúde e na redução das desigualdades alimentares e nutricionais. Objetivou-se analisar a tendência temporal dos marcadores do consumo alimentar de adultos cadastrados no Sistema de Vigilância Alimentar e Nutricional no Brasil e nas macrorregiões no período 2015-2019 e comparar o consumo de 2015-2019 com o de 2020-2023.

## Métodos

### Delineamento 

Trata-se de estudo de análise de série temporal, com base nos dados secundários anuais dispostos na plataforma *online* do Sistema de Vigilância Alimentar e Nutricional versão Web, referente ao período 2015-2019, e estudo comparativo do período 2015-2019 com 2020-2023, analisando o Brasil e suas cinco macrorregiões. 

### Contexto

Os dados analisados foram consultados nos relatórios de acesso público (8), disponíveis no endereço eletrônico https://sisaps.saude.gov.br/sisvan/relatoriopublico/index, com registros relativos aos marcadores do consumo alimentar de indivíduos adultos nos períodos 2015-2019 e 2020-2023. 

A primeira versão, nomeada como Sistema de Vigilância Alimentar e Nutricional Módulo Municipal, foi disponibilizada pela Portaria nº 2.246, de 18 de outubro de 2004 (9). Em 2008, a nova versão foi disponibilizada por meio do Sistema de Vigilância Alimentar e Nutricional versão Web. Em 2015, os novos formulários de avaliação do consumo alimentar foram disponibilizados (4). Em 2017, o sistema lançou a versão 3.0 (10), com o objetivo de otimizar a integração do Sistema de Vigilância Alimentar e Nutricional com o e-SUS Atenção Básica, conforme a nota técnica do Ministério da Saúde (11). 

A escolha do período 2015-2019 deve-se ao fato de que 2015 foi o ano de introdução dos novos formulários de consumo alimentar, e 2019, o último ano antes do início da pandemia de covid-19. Optou-se por esse período considerando que, até 2019, não haviam sido implementadas medidas de controle e prevenção da doença, as quais, sabe-se, influenciaram os hábitos cotidianos e o acesso regular aos serviços de saúde. Pressupõe-se que os dados referentes a esse período representem o perfil habitual da população-alvo.

Além de verificar a tendência temporal dos valores anuais entre 2015 e 2019, investigou-se o consumo alimentar no período anterior e posterior ao início da pandemia, por meio de comparações entre os percentuais de consumo agregados nos períodos 2015-2019 e 2020-2023.

### Participantes

Foram analisados dados agregados por região geográfica (macrorregiões e Brasil), oriundos de indivíduos adultos de 20-59 anos de ambos os sexos com dados disponíveis no Sistema de Vigilância Alimentar e Nutricional versão Web.

### Variáveis 

Os novos formulários dos marcadores de consumo alimentar do Sistema de Vigilância Alimentar e Nutricional, introduzidos em 2015, foram elaborados com base na nova proposta do guia alimentar. Contemplou-se o consumo alimentar de crianças maiores de 2 anos, adolescentes, adultos, idosos e gestantes, e avaliaram-se os alimentos consumidos no dia anterior, o que contribuiu para minimizar possíveis vieses de memória (2-4). 

O estudo analisou marcadores do consumo alimentar (saudáveis ou não saudáveis) do dia anterior. Considerou-se como comportamento saudável o consumo de feijão, frutas frescas, verduras e legumes; e não saudável o de hambúrguer e embutidos, bebidas adoçadas, macarrão instantâneo, salgadinhos de pacote ou biscoitos salgados, bem como o consumo de biscoitos recheados, doces ou guloseimas (4).

### Fontes de dados e mensuração

Os dados foram extraídos da plataforma do Sistema de Vigilância Alimentar e Nutricional, acessada por meio do endereço eletrônico https://sisaps.saude.gov.br/sisvan/relatoriopublico/index. Na plataforma, estavam dispostos os relatórios públicos anuais, que contemplam todos os tipos de acompanhamentos registrados, oriundos do próprio sistema, do sistema de gestão do Programa Bolsa Família e do e-SUS Atenção Primária à Saúde (8). Para este estudo, foram extraídos dados de consumo alimentar da última avaliação do ano para cada indivíduo adulto, que foram coletados no referido sítio eletrônico e extraídos no formato de planilha em Excel em 20 de março de 2024. 

### Controle de viés

No intuito de reduzir o risco da ocorrência de vieses, foram aplicados a priori protocolos do Sistema de Vigilância Alimentar e Nutricional para exclusão de valores implausíveis. 

### Tamanho do estudo 

Foram analisados dados agregados por região geográfica (macrorregiões e Brasil), com 7.549.781 adultos registrados. O percentual de cobertura do Sistema de Vigilância Alimentar e Nutricional foi calculado pelo número anual de registros, dividido pela população usuária exclusiva do SUS, multiplicado por 100. A população usuária exclusiva do SUS foi estimada a partir do número de brasileiros de 20-59 anos (12), subtraído do número de brasileiros nessa faixa etária que eram beneficiários de planos de saúde privados com assistência médica em junho de cada ano entre 2015 e 2023, segundo os dados da Agência Nacional de Saúde Suplementar (13).

### Métodos estatísticos

A variação percentual no período de cada marcador do consumo alimentar entre 2015 e 2019 foi calculada por meio da aplicação da seguinte equação: “((Valor em 2019 / Valor em 2015) - 1) x 100”. Cálculo semelhante foi aplicado entre 2020 e 2023. A associação entre o incremento de um ano e o consumo alimentar por meio dos marcadores foi investigada mediante o modelo de regressão de Prais-Winsten, na qual a proporção do consumo dos marcadores foi utilizada como variável dependente. Essa variável passou por transformação logarítmica, e os coeficientes betas resultantes foram submetidos a uma função exponencial. A variação percentual anual foi calculada por meio da equação “(1 - e^β^) x 100”.

Os valores de 2020 a 2023 foram comparados com os valores de 2015 a 2019, a partir do cálculo de variação percentual. Esse cálculo consistiu na subtração dos percentuais de 2020 a 2023 pelos percentuais de 2015 a 2019, divididos pelo percentual de 2020 a 2023, com resultado multiplicado por 100. A comparação desses períodos foi realizada pela regressão de Prais-Winsten. Variações percentuais anuais positivas ou negativas indicaram tendências crescentes ou decrescentes.

Para todas as análises, considerou-se significativo p-valor<0,05. As análises estatísticas foram realizadas com o uso do software Stata Statistical: versão 17 (College Station, TX: StataCorp LLC), utilizando a biblioteca “prais” (14).

### Acesso aos dados e métodos de limpeza

O processo realizado nesta pesquisa atendeu às normas do Conselho Nacional de Saúde, como também às normas e às instruções da Portaria nº 884, de 13 de dezembro de 2011 (15). O projeto foi aprovado pelo Comitê de Ética em Pesquisa da Universidade de Fortaleza. Todos os registros disponíveis no sistema para esses recortes etário, temporal e espacial foram utilizados nesta análise. 

### Pareamento dos dados

Para este estudo, não houve necessidade de vinculação de dados em quaisquer níveis ou bancos de dados. Tratou-se da utilização dos dados do Sistema de Vigilância Alimentar e Nutricional versão Web.

## Resultados

Entre 2015 e 2023, 7.549.781 adultos de 20-59 anos, de ambos os sexos, foram registrados pelo sistema. A cobertura anual do Sistema de Vigilância Alimentar e Nutricional em âmbito nacional apresentou o menor percentual em 2015 (0,2%). O ano de 2019 (1,0%) marcou o aumento dessa cobertura. Em 2020, houve queda (0,8%), aumentando de forma crescente em 2021 (1,2%), 2022 (1,8%) e 2023 (3,1%) (Tabela 1). 

**Tabela 1:** Percentual de cobertura nacional anual de adultos registrados Sistema de Vigilância Alimentar e Nutricional. Brasil, 2015-2023

Ano	(%)
2015	0,2
2016	0,4
2017	1,0
2018	0,8
2019	1,0
2020	0,8
2021	1,2
2022	1,8
2023	3,1

A variação percentual anual de consumo alimentar de feijão se manteve estável no Brasil e nas macrorregiões entre 2015 e 2019. Nesses cinco anos, o percentual de pessoas registradas que consumiram frutas frescas no dia anterior no Brasil cresceu, em média, 1,4% ao ano (intervalo de confiança de 95% [IC95%] 0,3; 2,6). O consumo de hambúrguer e embutidos cresceu 4,5% ao ano (IC95% 2,3; 6,7), sendo 1,2% menor para bebidas adoçadas (IC95% -1,8; -0,6). Na região Norte, houve redução de 4,5% ao ano no consumo de bebidas adoçadas (IC95% -6,8; -2,3) e de 6,4% (IC95% -12,1; -0,3) de biscoitos recheados, doces ou guloseimas (Tabela 2). 

**Tabela 2:** Variação percentual anual e intervalos de confiança de 95% (IC95%) dos marcadores do consumo alimentar do Sistema de Vigilância Alimentar e Nutricional. Brasil e regiões geográficas, 2015-2019

Marcadores	Variação percentual no período (%)^a^	Variação percentual anual^b^		
		(%)	(IC95%)	Tendência
Brasil				
Feijão	0,0	0,2	(-0,4; 0,8)	Estável
Frutas frescas	2,8	1,4	(0,3; 2,6)	Crescente
Verduras e legumes	4,1	1,5	(-0,7; 3,7)	Estável
Hambúrguer e embutidos	15,6	4,5	(2,3; 6,7)	Crescente
Bebidas adoçadas	-5,3	-1,2	(-1,8; -0,6)	Decrescente
Macarrão instantâneo etc.	-5,7	-0,2	(-5,9; 5,7)	Estável
Biscoitos recheados etc.	-2,8	0,1	(-1,1; 1,3)	Estável
Norte				
Feijão	-6,3	-0,2	(-2,3; 2,0)	Estável
Frutas frescas	-4,2	-0,4	(-1,7; 0,9)	Estável
Verduras e legumes	0,0	0,5	(-0,8; 1,9)	Estável
Hambúrguer e embutidos	-6,9	1,2	(-5,6; 8,4)	Estável
Bebidas adoçadas	-17,0	-4,5	(-6,8; -2,3)	Decrescente
Macarrão instantâneo etc.	-23,0	-4,8	(-13,3; 4,6)	Estável
Biscoitos recheados etc.	-25,0	-6,4	(-12,1; -0,3)	Decrescente
Nordeste				
Feijão	0,0	0,1	(-1,1; 1,4)	Estável
Frutas frescas	2,8	1,5	(-0,6; 3,6)	Estável
Verduras e legumes	9,1	3,0	(1,4; 4,5)	Crescente
Hambúrguer e embutidos	3,3	1,6	(-7,3; 11,4)	Estável
Bebidas adoçadas	-2,0	-0,6	(-5,4; 4,5)	Estável
Macarrão instantâneo etc.	-10,5	-2,0	(-6,9; 3,1)	Estável
Biscoitos recheados etc.	3,2	1,1	(-0,0; 2,2)	Estável
Centro-Oeste				
Feijão	3,4	1,1	(-2,1; 4,5)	Estável
Frutas frescas	-1,3	0,4	(-2,0; 2,8)	Estável
Verduras e legumes	3,8	1,2	(0,3; 2,2)	Crescente
Hambúrguer e embutidos	20,6	5,0	(-4,4; 15,4)	Estável
Bebidas adoçadas	-1,7	-0,1	(-6,3; 6,4)	Estável
Macarrão instantâneo etc.	-13,9	-2,9	(-15,6; 11,8)	Estável
Biscoitos recheados etc.	-12,2	-3,1	(-6,4; 0,4)	Estável
Sudeste				
Feijão	2,3	0,7	(-0,3; 1,8)	Estável
Frutas frescas	2,9	1,5	(0,4; 2,6)	Crescente
Verduras e legumes	2,6	0,8	(-0,3; 2,0)	Estável
Hambúrguer e embutidos	18,2	3,9	(1,2; 6,7)	Crescente
Bebidas adoçadas	-8,2	-1,4	(-2,4; -0,4)	Decrescente
Macarrão instantâneo etc.	-3,1	0,6	(-1,4; 2,6)	Estável
Biscoitos recheados etc.	-7,7	-2,2	(-2,9; -1,6)	Decrescente
Sul				
Feijão	-2,5	-1,1	(-3,6; 1,5)	Estável
Frutas frescas	4,1	0,9	(-3,0; 5,0)	Estável
Verduras e legumes	-1,2	-0,4	(-3,5; 2,9)	Estável
Hambúrguer e embutidos	17,8	4,6	(-2,5; 12,3)	Estável
Bebidas adoçadas	-1,6	-1,1	(-1,2; -1,1)	Decrescente
Macarrão instantâneo etc.	9,4	3,8	(1,0; 6,6)	Crescente
Biscoitos recheados etc.	11,1	3,7	(1,9; 5,5)	Crescente

^a^Variação percentual dos marcadores do consumo alimentar no período 2015-2019; ^b^Variação percentual anual dos marcadores do consumo alimentar.

Entre 2015 e 2019, na região Nordeste, o percentual de pessoas que consumiram verduras e legumes no dia anterior cresceu, em média, 3,0% por ano (IC95% 1,4; 4,5). Na região Sudeste, o percentual de pessoas registradas que consumiram frutas no dia anterior cresceu, em média, 1,5% ao ano (IC95% 0,4; 2,6). O consumo de hambúrguer e embutidos no dia anterior também cresceu, sendo 3,9% ao ano (IC95% 1,2; 6,7). Houve decréscimo de 1,4% das bebidas adoçadas (IC95% -2,4; -0,4) e de 2,2% (IC95% -2,9; -1,6) ao ano de biscoitos recheados, doces e guloseimas (Tabela 2). 

Na região Sul, entre 2015 e 2019, o percentual de pessoas que consumiram bebidas adoçadas no dia anterior diminuiu, em média, 1,1% ao ano (IC95% -1,2; -1,1). O consumo de macarrão instantâneo, salgadinhos de pacote ou biscoitos salgados apresentou crescimento, em média, de 3,8% ao ano (IC95% 1,0; 6,6), e de biscoitos recheados, doces e guloseimas o crescimento de 3,7% ao ano (IC95% 1,9; 5,5). No Centro-Oeste, o percentual de pessoas registradas que consumiram verduras e legumes no dia anterior cresceu, em média, 1,2% ao ano (IC95% 0,3; 2,2) entre 2015 e 2019 (Tabela 2). 

Comparando os dois períodos, houve evidência estatística de crescimento de marcadores não saudáveis do consumo alimentar em todas as macrorregiões e no Brasil no período 2020-2023, quando comparado a 2015-2019. Nas regiões Norte, Nordeste, Centro-Oeste e Sudeste e no Brasil, houve aumento do consumo de hambúrguer e embutidos. Nas regiões Centro-Oeste, Sudeste e Sul e no Brasil, houve aumento do consumo de macarrão instantâneo, salgadinhos de pacote e biscoitos salgados. Para o Brasil e as regiões Norte, Nordeste, Sul e Sudeste, não houve aumento ou redução para os demais alimentos não saudáveis. No Centro-Oeste, houve aumento do consumo de bebidas adoçadas e de biscoitos recheados, doces e guloseimas (Tabela 3).

**Tabela 3:** Percentual e variação percentual dos marcadores do consumo alimentar do Sistema de Vigilância Alimentar e Nutricional. Brasil e regiões geográficas, 2015-2019 e 2020-2023

Marcadores	Percentual no período 2015-2019	Percentual no período 2020-2023	Variação percentual entre os períodos	p-valor
Brasil				
Feijão	85,3	80,2	-6,0	0,142
Frutas frescas	73,0	71,4	-2,3	0,365
Verduras e legumes	76,2	79,1	3,8	0,072
Hambúrguer e embutidos	35,6	42,3	18,8	0,006
Bebidas adoçadas	54,6	54,8	0,5	0,620
Macarrão instantâneo etc.	32,1	34,0	5,8	0,041
Biscoitos recheados etc.	35,0	36,4	3,9	0,116
Norte				
Feijão	76,4	75,5	-1,2	0,290
Frutas frescas	68,3	73,0	6,8	0,979
Verduras e legumes	65,6	76,2	16,2	0,003
Hambúrguer e embutidos	26,5	36,1	36,0	<0,001
Bebidas adoçadas	52,0	51,7	-0,5	0,863
Macarrão instantâneo etc.	31,5	31,2	-1,2	0,648
Biscoitos recheados etc.	31,8	32,6	2,5	0,868
Nordeste				
Feijão	88,9	84,7	-4,7	0,154
Frutas frescas	72,9	74,3	1,9	0,872
Verduras e legumes	69,4	76,1	9,7	0,005
Hambúrguer e embutidos	28,6	37,0	29,2	0,003
Bebidas adoçadas	48,3	48,9	1,3	0,493
Macarrão instantâneo etc.	33,9	34,1	0,6	0,766
Biscoitos recheados etc.	31,1	32,3	3,9	0,062
Centro-Oeste				
Feijão	87,9	82,3	-6,4	0,211
Frutas frescas	72,8	70,0	-4,5	0,252
Verduras e legumes	80,7	82,8	2,7	0,035
Hambúrguer e embutidos	35,4	47,2	33,2	0,002
Bebidas adoçadas	55,9	61,0	9,1	0,021
Macarrão instantâneo etc.	28,6	34,8	21,6	0,020
Biscoitos recheados etc.	35,1	40,9	16,4	0,019
Sudeste				
Feijão	87,9	85,3	-3,0	0,292
Frutas frescas	71,8	72,3	0,7	0,629
Verduras e legumes	80,0	83,9	5,0	0,002
Hambúrguer e embutidos	37,4	44,7	19,5	0,001
Bebidas adoçadas	57,7	58,8	1,9	0,601
Macarrão instantâneo etc.	31,3	33,4	6,6	0,025
Biscoitos recheados etc.	36,8	38,5	4,6	0,276
Sul				
Feijão	78,5	61,5	-21,7	0,041
Frutas frescas	79,5	61,9	-22,2	0,062
Verduras e legumes	81,2	75,1	-7,6	0,020
Hambúrguer e embutidos	47,9	52,3	9,3	0,091
Bebidas adoçadas	58,4	57,7	-1,2	0,908
Macarrão instantâneo etc.	33,2	37,4	12,8	0,007
Biscoitos recheados etc.	39,2	41,6	6,2	0,053

Não houve modificação no país em relação aos marcadores de consumo saudáveis, mas, na análise estratificada por regiões, houve crescimento significativo de verduras/legumes em todas as regiões, exceto no Sul, onde houve redução. No Sul, houve redução significativa no consumo de feijão (Tabela 3).

## Discussão

Este estudo analisou a tendência temporal do consumo alimentar de adultos registrados no Sistema de Vigilância Alimentar e Nutricional no período 2015-2019 e em comparação com 2020-2023. No Brasil, observou-se tendência temporal de crescimento do consumo de hambúrguer e embutidos e frutas frescas e redução de bebidas adoçadas. A comparação entre os períodos apontou possíveis modificações do consumo alimentar devido à pandemia de covid-19, com aumento no consumo de hambúrguer e embutidos e macarrão instantâneo. 

A escolha dos marcadores, como instrumento de análise, permitiu identificar o consumo de alimentos saudáveis e não saudáveis de indivíduos adultos que são usuários da atenção primária e que são atendidos no SUS no Brasil e nas suas regiões. Os marcadores saudáveis contemplam o consumo de feijão, frutas frescas, verduras e legumes; e os não saudáveis se relacionam ao de hambúrguer e embutidos, bebidas adoçadas, macarrão instantâneo, salgadinhos de pacote ou biscoitos salgados, bem como os biscoitos recheados, doces ou guloseimas (4). Tal instrumento tornou-se ferramenta para designar condições de saúde, orientar práticas alimentares e retratar fragilidades associadas à dieta, funcionando de forma efetiva e objetiva como marcadores (5).

Os resultados encontrados devem ser interpretados considerando algumas limitações. Por ser um estudo ecológico, pode possuir viés devido às inferências causais. Considerando a qualidade dos dados em sistemas de informação em saúde, em uma perspectiva multidimensional, avaliou-se a cobertura dos dados inseridos, por tratar-se de uma das dimensões mais avaliadas no Sistema de Vigilância Alimentar e Nutricional e em outros sistemas (16). Ainda que apresente modesta cobertura em comparação com os dados antropométricos, há um perfil de crescimento consistente (17). Os formulários avaliam os alimentos que foram consumidos no dia anterior, amenizando vieses de memória, com questões objetivas, claras e de simples aplicação. Tal instrumento é recomendado pelo Ministério da Saúde. Não há informações sobre quantidades e frequências habituais. 

Entre os percentuais de cobertura em âmbito nacional, o menor percentual apresentado em 2015 era esperado, pois marcou o início da implementação dos novos formulários do consumo alimentar. Entre 2018 e 2019, foi notório o aumento dessa cobertura, quando a entrada de dados se iniciou via e-SUS Atenção Básica, em detrimento do Sistema de Vigilância Alimentar e Nutricional versão *Web* em 2017. O ano de 2019 foi marcado como o último antes do início da pandemia de covid-19, com o melhor percentual entre os anos anteriores. Em 2020, ocorreu o declínio desse percentual na cobertura. Os anos que se sucedem à pandemia continuaram em crescimento dessa cobertura. 

O sistema vem sendo aperfeiçoado e atualizado ao longo dos anos, melhorando a sua cobertura e a qualidade dos dados. A cobertura populacional total foi considerada preambular, apresentando algumas limitações à representatividade dos dados. Foram apontados avanços na cobertura do sistema e tendência crescente com a entrada dos dados via e-SUS Atenção Primária ao longo dos anos (18,19).

Comparando as regiões brasileiras entre 2015 e 2019, tanto a nível Brasil como macrorregiões, foi identificada a variação estável no percentual anual do marcador de consumo alimentar de feijão. Houve maior crescimento no percentual de marcadores saudáveis na região Nordeste para verduras e legumes. A maior diminuição de alimentação não saudável ocorreu na região Norte. O maior incremento percentual de não saudáveis foi observado na região Centro-Oeste, na proporção de alimentos relativos a carnes processadas, como hambúrguer e embutidos.

A ingestão de frutas, verduras e legumes foi protetor para as doenças cardiovasculares, sendo recomendada a ingestão diária desses alimentos (3). Considerou-se a frequência estável entre 2008 e 2021 de adultos que consumiram regularmente frutas e hortaliças, variando de 33,0%, em 2008, a 34,2%, em 2021 (20).

Os alimentos processados e ultraprocessados que foram mais consumidos pela amostra deste estudo, entre 2015 e 2019, foram hambúrguer e embutidos no Brasil e na região Sudeste e macarrão instantâneo, salgadinhos de pacote ou biscoitos salgados e biscoitos recheados, doces ou guloseimas na região Sul. O decréscimo observado no percentual de consumidores, no Brasil e nas regiões Norte, Sudeste e Sul, no consumo de bebidas adoçadas se coadunou com dados anteriores (21,22). 

No Brasil, houve a maior participação dos ultraprocessados na dieta, estando relacionado aos agravos em saúde na população adulta, o que caracterizava o cenário acerca do perfil nutricional (23-25). Acentuou-se que o uso dos marcadores, a nível individual e populacional, possibilitou o reconhecimento de hábitos alimentares saudáveis e não saudáveis, além de permitir o acompanhamento da qualidade da alimentação de toda a população brasileira (6,7).

Este estudo observou que, entre os recortes temporais de 2015-2019 e 2020-2023, houve aumento de marcadores não saudáveis em todas as macrorregiões e no Brasil. A pandemia de covid-19 modificou os hábitos alimentares da população, observando-se redução no consumo de alimentos in natura e minimamente processados e incremento no de ultraprocessados (26,27). Foram evidentes associações entre variáveis socioeconômicas, de estilo de vida e hábitos alimentares inadequados (26) juntamente com comportamentos de estresse, ansiedade e depressão durante o período pandêmico (28,29).

Entre o consumo nos períodos 2015-2019 e 2020-2023, destacou-se, entre os alimentos saudáveis, os maiores percentuais de variação de verduras e legumes nas regiões Nordeste (+9,7%) e Norte (+16,2%) e dos não saudáveis na região Centro-Oeste, de hambúrguer e embutidos (+33,2%), bebidas adoçadas (+9,1%), macarrão instantâneo, salgadinhos de pacote ou biscoitos salgados (+21,6%) e de biscoitos recheados, doces ou guloseimas (+16,4%). Observou-se aumento de alimentos ultraprocessados pela população adulta brasileira durante a pandemia de covid-19 (26,27).

O consumo de feijão não sofreu variação nos dois recortes temporais, com exceção da região Sul, onde houve redução. Esse dado diferiu das informações obtidas anteriormente. Estas indicavam que a frequência de adultos, entre 2007 e 2021, que referiram a ingestão de feijão em cinco ou mais dias da semana diminuiu, variando de 66,8%, em 2007, a 60,4% em 2021 (20). Dados da pesquisa de orçamento familiar em 2008-2009 (30) e 2017-2018 (22) também retrataram o declínio do feijão na dieta brasileira. 

Pode-se compreender que o consumo alimentar de usuários adultos da atenção primária no Brasil possui disparidades regionais, acentuando o reflexo das características climáticas, atividades econômicas, acesso geográfico, diversidade de culturas alimentares, além de desigualdades sociais entre as regiões (18,19). Isso contribui para as mudanças no padrão alimentar, associando-se aos comportamentos saudáveis ou não saudáveis encontrados neste estudo. 

O sistema tem contribuído para o monitoramento e a gestão das informações da Vigilância Alimentar e Nutricional, conforme as diretrizes da Política Nacional de Alimentação e Nutrição (31). As informações retiradas dos marcadores abrangem o diagnóstico e a vigilância das condições alimentares da população adulta, que avaliam a eficácia e a eficiência das políticas públicas (6,16). O uso de dados secundários, analisados por meio do Sistema de Vigilância Alimentar e Nutricional, é modesto nesta população, constituindo amplo espaço de pesquisas futuras. 

Conclui-se que houve aumento na cobertura anual de indivíduos adultos no Brasil com registros de marcadores do consumo alimentar, que alcançou, em 2019, 1,0% da população no período pré-pandêmico. Houve queda dessa cobertura no ano seguinte e aumento nos anos posteriores. 

Os resultados encontrados identificaram consumo alimentar nacional progressivamente não saudável no período 2015-2019, principalmente com a presença de consumo aumentado de hambúrguer e embutidos e de macarrão instantâneo, salgadinhos de pacote ou biscoitos salgados. Essa tendência encontrada pode ser contrabalançada pela presença aumentada de verduras/legumes na alimentação consumida. A comparação realizada entre os períodos 2015-2019 e 2020-2023 apontou possíveis modificações de consumo alimentar devido à pandemia covid-19.

## Data Availability

O banco de dados utilizado na pesquisa está disponível em https://sisaps.saude.gov.br/sisvan/relatoriopublico/index.
